# Fibroblast drug scavenging increases intratumoural gemcitabine accumulation in murine pancreas cancer

**DOI:** 10.1136/gutjnl-2016-311954

**Published:** 2017-01-10

**Authors:** E Hessmann, M S Patzak, L Klein, N Chen, V Kari, I Ramu, T E Bapiro, K K Frese, A Gopinathan, F M Richards, D I Jodrell, C Verbeke, X Li, R Heuchel, J M Löhr, S A Johnsen, T M Gress, V Ellenrieder, A Neesse

**Affiliations:** 1 Department Gastroenterology and Gastrointestinal Oncology, University Medical Centre Goettingen, Goettingen, Germany; 2 Department of General, Visceral and Pediatric Surgery, University Medical Center Goettingen, Goettingen, Germany; 3 Cancer Research UK Cambridge Institute, The University of Cambridge, Li Ka Shing Centre, Cambridge, UK; 4 Oncology iMED DMPK AstraZeneca UK Ltd, HODGKIN C/o B310 Cambridge Science Park, Cambridge, UK; 5 The University of Manchester, Cancer Research UK Manchester Institute, Manchester, UK; 6 Department of Oncology, University of Cambridge, Cambridge, UK; 7 Department of Pathology, Karolinska University Hospital, Stockholm, Sweden; 8 Department of Pathology, Institute of Clinical Medicine, University of Oslo, Oslo, Norway; 9 Department of Clinical Science, Intervention and Technology (CLINTEC), Karolinska Institutet and Center for Digestive Diseases, Karolinska University Hospital, Stockholm, Sweden; 10 Department of Gastroenterology, Endocrinology and Metabolism, Philipps University Marburg, Marburg, Germany

**Keywords:** PANCREATIC CANCER, DRUG METABOLISM, CHEMOTHERAPY, PANCREATIC FIBROSIS

## Abstract

**Objective:**

Desmoplasia and hypovascularity are thought to impede drug delivery in pancreatic ductal adenocarcinoma (PDAC). However, stromal depletion approaches have failed to show clinical responses in patients. Here, we aimed to revisit the role of the tumour microenvironment as a physical barrier for gemcitabine delivery.

**Design:**

Gemcitabine metabolites were analysed in *LSL-Kras^G12D/+^*; *LSL-Trp53^R172H/+^*; *Pdx-1-Cre* (KPC) murine tumours and matched liver metastases, primary tumour cell lines, cancer-associated fibroblasts (CAFs) and pancreatic stellate cells (PSCs) by liquid chromatography-mass spectrometry/mass spectrometry. Functional and preclinical experiments, as well as expression analysis of stromal markers and gemcitabine metabolism pathways were performed in murine and human specimen to investigate the preclinical implications and the mechanism of gemcitabine accumulation.

**Results:**

Gemcitabine accumulation was significantly enhanced in fibroblast-rich tumours compared with liver metastases and normal liver. In vitro, significantly increased concentrations of activated 2′,2′-difluorodeoxycytidine-5′-triphosphate (dFdCTP) and greatly reduced amounts of the inactive gemcitabine metabolite 2′,2′-difluorodeoxyuridine were detected in PSCs and CAFs. Mechanistically, key metabolic enzymes involved in gemcitabine inactivation such as hydrolytic cytosolic 5′-nucleotidases (Nt5c1A, Nt5c3) were expressed at low levels in CAFs in vitro and in vivo, and recombinant expression of Nt5c1A resulted in decreased intracellular dFdCTP concentrations in vitro. Moreover, gemcitabine treatment in KPC mice reduced the number of liver metastases by >50%.

**Conclusions:**

Our findings suggest that fibroblast drug scavenging may contribute to the clinical failure of gemcitabine in desmoplastic PDAC. Metabolic targeting of CAFs may thus be a promising strategy to enhance the antiproliferative effects of gemcitabine.

**Significance of this study**

**What is already known on this subject?**

- Pancreatic ductal adenocarcinoma (PDAC) and liver metastases are characterised by pronounced tumour stroma consisting of activated cancer-associated fibroblasts (CAFs), and abundant extracellular matrix components.
- Depletion of stromal components by various pharmacological approaches has resulted in increased intratumoural gemcitabine levels suggesting that the tumour stroma may act as physical barrier for drug delivery.
- Depletion of CAFs has been shown to promote tumour growth and spread.

**What are the new findings?**

- Primary human and murine pancreatic tumours show an increased number of α-smooth muscle actin-positive fibroblasts compared with matched liver metastases. Murine primary tumours accumulate significantly more gemcitabine metabolites.
- In CAFs and pancreatic stellate cells (PSCs), significantly higher intracellular concentrations of gemcitabine metabolites were identified, in comparison to tumour cell lines derived from primary murine PDAC and metastases.
- Lower expression of key inactivating enzymes, such as hydrolytic cytosolic 5′-nucleotidases in CAFs and PSCs increase the intracellular concentration of gemcitabine when compared with cancer cells and may serve as targets for stromal reprogramming.

**How might it impact on clinical practice in the foreseeable future?**

- CAFs and PSCs entrap active gemcitabine intracellularly and may thus limit the availability of the drug for cancer cells.
- Targeting the metabolic machinery of fibroblasts may enhance local availability of gemcitabine in the tumour without interfering with the tumour-restraining properties of CAFs.

## Introduction

Pancreatic ductal adenocarcinoma (PDAC) is considered one of the most aggressive solid tumours with increasing incidence worldwide.^1^ By 2030, PDAC is projected to become the second leading cause of cancer-related deaths following lung cancer in the USA.^2^ Over several decades, gemcitabine has remained the standard of care chemotherapy despite only marginal effects on patient survival.^3^ More recently, FOLFIRINOX (oxaliplatin, irinotecan, leucovorin and 5-fluorouracil (5-FU)) and nab-paclitaxel+gemcitabine have extended the panel of available chemotherapies, for the first time achieving significant survival benefits for patients with metastatic PDAC.^4^ ^5^ However, increased rates of toxicity often limit the frequent clinical use of both regimens in patients with PDAC. More than any other solid cancer, PDAC is characterised by abundant tumour stroma with activated cancer-associated fibroblasts (CAFs) that maintain a dense biophysical meshwork around neoplastic ductal cells consisting of components such as hyaluronic acid, fibronectin, secreted protein acidic and rich in cysteine (SPARC) and collagens.^6^ Besides the complex biochemical cancer-stroma crosstalk,^7–9^ the distinct stromal architecture has been postulated to create physical barriers for drug delivery,^10^ and sparked a new era of stromal depletion approaches.^11^

Indeed, subsequent preclinical investigations introduced alternative strategies to successfully deplete stromal fibrosis and relieve vessel compression. For instance, degradation of hyaluronan and collagen, as well as reduced fibrosis following vitamin-D receptor activation in pancreatic stellate cells (PSCs) resulted in increased drug delivery and response to gemcitabine treatment in various genetically engineered mouse models (GEMMs).^12–15^ Furthermore, some studies reported stromal ablation on nab-paclitaxel administration in mice and humans suggesting intratumoural nab-paclitaxel sequestration via binding of stromal SPARC to albumin-coated paclitaxel.^16^ ^17^ However, others have not observed stromal depletion on nab-paclitaxel,^18–20^ and a recent evaluation of 256 patients from a large phase III clinical trial did not show a correlation of stromal SPARC expression and overall survival on nab-paclitaxel+gemcitabine treatment.^21^ Consequently, the concept of the stroma acting as physical barrier to prevent gemcitabine and other therapeutic molecules to enter the tumour has been challenged recently. Studies in patients with PDAC using CT-derived transport properties showed significant interpatient and intratumoural heterogeneity of gemcitabine incorporation into the DNA despite similar intravascular pharmacokinetics. Notably, stromal content correlated with gemcitabine incorporation only after accounting for levels of human equilibrative nucleoside transporter 1 (hENT1).^22^ ^23^ Further preclinical and clinical data have confirmed the observation that levels of the gemcitabine transporter hENT1, the activating enzyme deoxycytidine kinase (dCK) and the inactivating enzyme cytidine deaminase (Cda) correspond with survival in patients and preclinical response to gemcitabine,^18^ ^24–26^ suggesting that gemcitabine metabolism rather than biophysical properties matter most. Moreover, limitations of past experimental studies assessing drug delivery in murine pancreatic tumours included the use of different analytical methods such as nuclear MRI (^19^F NMR) and liquid chromatography-mass spectrometry/mass spectrometry (LC-MS/MS). Both methods cannot be compared directly as ^19^F NMR is unable to distinguish all gemcitabine metabolites, and is less sensitive.^27^ Vascular function and drug delivery studies with doxorubicin and fluorophore-labelled lectin could only assess very early time-points after drug administration (∼5 min), thus capturing a snapshot of intratumoural drug accumulation that may not be representative of the genuine pharmacokinetic and pharmacodynamic properties of gemcitabine. Thus, the relevance of vessel patency and mean vessel density (MVD) for gemcitabine delivery may have been overestimated using these short-time assays. Notably, PDAC tissue analysis was limited to the tumour bulk, and consequently distinction between tumour and stromal cells was not possible.

In order to overcome these limitations, we recently analysed the pharmacokinetic and pharmacodynamic profile of gemcitabine in *LSLKras^G12D/+^; LSL-Trp53^R172H/+^; Pdx-1-Cre* (KPC) tumours over time using LC-MS/MS as the most sensitive detection method for gemcitabine metabolites. Whereas serum concentrations of the gemcitabine prodrug 2′,2′-difluorodeoxycytidine (dFdC) peaked 1 hour after intraperitoneal injection, intratumoural accumulation of the activated gemcitabine metabolite 2′,2′-difluorodeoxycytidine-5′-triphosphate (dFdCTP) reached maximum levels only after 2 hours and coincided with the maximum induction of apoptotic cell death as evidenced by cleaved caspase-3 immunohistochemistry.^28^

Using this standardised protocol, we here revisit the pharmacokinetic profile of gemcitabine in murine PDAC in vitro and vivo, and dissect the stromal and the neoplastic compartment of primary tumours and matched liver metastases. Our findings offer an alternative explanation for the clinical failure of gemcitabine in stroma-rich PDAC.

## Materials and methods

For details, see online supplementary material and methods.

10.1136/gutjnl-2016-311954.supp1 supplementary material and methods

### Human pancreatic cancer specimen

PDAC tumour samples with matched liver metastases were obtained from formalin-fixed and paraffin-embedded (FFPE) tissue blocks collected for clinical purposes at Karolinska Institute. The PDAC diagnosis was confirmed by a staff pathologist at Karolinska Institute. Ethical approval was obtained from Karolinska Institutional Review Board (EPN D-No. 2014/2147-31/1). Tissue microarrays with PDAC (n=50) were assembled from representative FFPE archival tissue blocks that had been sampled from pancreatoduodenectomy specimens at Oslo University Hospital, Norway. Each tumour was represented by two 1.0 mm cores. Permission for the study was obtained from the Regional Committee for Medical and Health Research Ethics for Southern Norway (REK nr. S-05081).

### Preclinical mouse studies

For pharmacokinetic studies, gemcitabine was administered at 100 mg/kg by intraperitoneal injections. Tissues were harvested 2 hours after gemcitabine administration.

### Liquid chromatography-mass spectrometry/mass spectrometry

#### Gemcitabine (dFdC, 2′,2′-difluorodeoxyuridine, dFdCTP) and 5-FU

Fresh frozen tumour samples were processed and analysed using LC-MS/MS as previously described.^27^ Briefly, LC-MS/MS for gemcitabine and metabolites was performed on a TSQ Vantage triple stage quadrupole mass spectrometer (Thermo Fisher Scientific, USA) fitted with a heated electrospray ionisation II probe operated in positive and negative mode at a spray voltage of 2.5 kV, capillary temperature of 150°C. Quantitative data acquisition was done using LC Quan2.5.6 (Thermo Fisher Scientific). LC-MS/MS for 5-FU was performed by the CRUK Cambridge Institute Pharmacokinetics & Bioanalytics (PKB) Core Facility, as previously described,^29^ but using a Sciex 6500 Triple Quad mass spectrometer with electrospray ionisation at 500°C.

### Statistical analysis

Statistical analysis was carried out using GraphPad Prism V.6.05 (GraphPad Software). The Mann-Whitney non-parametric U test was used unless indicated otherwise, and results are presented as mean±SE; p<0.05 was considered to be statistically significant.

## Results

### Gemcitabine accumulation is increased in KPC tumours compared with liver metastases and normal liver tissue but does not correlate with survival

**Figure 1:**
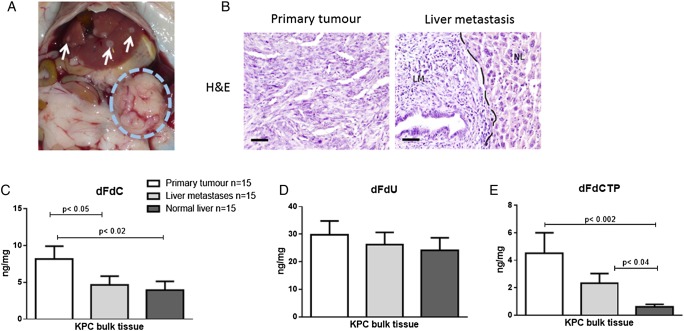
Pharmacokinetic profile of gemcitabine metabolites in primary pancreatic tumours, liver metastases and normal liver tissue in corresponding KPC mice. (A) Necropsy view of a KPC mouse with a large pancreatic tumour (dotted blue circle) and several liver metastases (white arrows). (B) H&E staining of a representative KPC tumour with matched liver metastasis. NL, normal liver, LM, liver metastasis. Scale bars, 50 µm. (C) Tumour-bearing KPC mice were treated with one dose of gemcitabine at 100 mg/kg intraperitoneally. Tumour tissues, liver metastases and normal liver tissue were excised and assessed for gemcitabine metabolites 2 hours later by liquid chromatography-mass spectrometry/mass spectrometry (n=15). Gemcitabine is significantly elevated in primary tumours compared with liver metastases (p<0.05) and normal liver tissue (p<0.02). (D) The deaminated and inactive metabolite 2′,2′-difluorodeoxyuridine (dFdU) shows no significant differences among the three groups. (E) The triple phosphorylated active gemcitabine metabolite 2′,2′-difluorodeoxycytidine-5′-triphosphate (dFdCTP) is significantly increased in primary pancreatic tumours as compared with normal liver tissue (p<0.01).

To determine whether overall survival in KPC mice is associated with intratumoural concentrations of gemcitabine, in particular the active, cytotoxic metabolite, we re-analysed n=10 KPC bulk tumours from a previously published preclinical trial^28^ for dFdCTP 2 hours after the last gemcitabine dose. Interestingly, there was no correlation of intratumoural dFdCTP amount and survival of KPC mice (Pearson's r=0.23) suggesting that active gemcitabine in bulk tumour tissue might not be a suitable predictor of response to treatment (see online supplementary figure S1A). For the combined primary tumour and liver metastasis study, we dosed tumour-bearing KPC mice with 100 mg/kg gemcitabine intraperitoneally. At this point, all mice had large pancreatic tumours with metastatic disease corresponding to stage IV pancreatic cancer in patients (figure 1A,B). As determined earlier,^28^ peak tumour concentrations of gemcitabine are reached 2 hours after intraperitoneal injection. Therefore, all mice (n=15) were sacrificed exactly 2 hours after gemcitabine administration, and fresh frozen samples from liver metastases, primary tumours and healthy liver tissue were immediately harvested. Using LC-MS/MS, we analysed the concentration of gemcitabine dFdC, the inactive metabolite 2′,2′-difluorodeoxyuridine (dFdU) and the active, cytotoxic gemcitabine metabolite dFdCTP using a previously established protocol.^27^ Surprisingly, the mean gemcitabine concentration was significantly higher in primary pancreatic tumours compared with liver metastases (8.1 ng/mg, 95% CI 4.4 to 11.8 vs 4.6 ng/mg, 95% CI 2.0 to 7.2; p<0.05) (figure 1C). The inactive metabolite dFdU did not significantly differ among the groups (figure 1D). Mean dFdCTP concentrations were not significantly elevated in primary tumours compared with liver metastases (4.5 ng/mg, 95% CI 1.3 to 7.7 vs 2.3 ng/mg 95% CI 0.8 to 3.8; not significant), but mean dFdCTP concentration was highly significantly elevated in primary tumours compared with corresponding normal liver specimen (4.5 ng/mg, 95% CI 1.3 to 7.7 vs 0.59 ng/mg, 95% CI 0.15 to 1.0; p<0.002) (figure 1E).

### Primary murine and human pancreatic tumours feature higher stromal content than matched liver metastases

**Figure 2:**
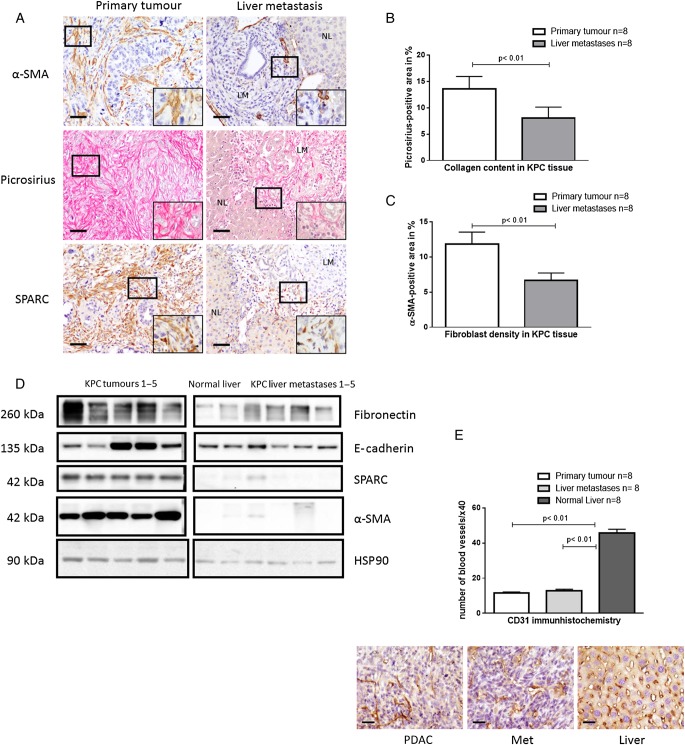
Primary tumours display higher stromal content than liver metastases. (A) Representative pictures of collagen, secreted protein acidic and rich in cysteine (SPARC) and α-smooth muscle actin (α-SMA) stains from primary murine pancreatic tumours and matched liver metastases (LM) with adjacent normal liver (NL) reveal increased cellular and acellular desmoplasia in primary tumours. Scale bars, 50 µm. (B and C) Automated quantification of n=8 primary tumours and n=8 liver metastases reveal significant increase in collagen and α-SMA area in primary tumours (p<0.01; Wilcoxon matched-pairs signed-rank test). (D) Western blot analysis of whole tissue lysates from KPC primary tumours (n=5), liver metastases (n=5) and normal liver tissue (n=1) confirm higher α-SMA, SPARC and fibronectin levels compared with liver metastases and normal liver. HSP90, heat shock protein 90. (E) Immunohistochemical CD31 analysis reveals comparable mean vessel density (MVD) in primary tumours (n=8) and liver metastases (n=8), whereas normal liver tissue (n=8) featured significantly higher MVD (p<0.01; Wilcoxon matched-pairs signed-rank test). Scale bars, 25 µm. PDAC, pancreatic ductal adenocarcinoma.

We reasoned that either stromal composition or vascularisation of the different tumour tissues may account for the significantly different gemcitabine concentrations. To this end, we systematically assessed and compared the desmoplastic reaction of primary KPC tumours and matched liver metastases (n=8) by biochemical and immunohistochemical methods. Immunohistochemistry for α-smooth muscle actin (α-SMA), collagen and SPARC provided clear evidence that desmoplasia, in particular fibroblast density, was reduced in liver metastases compared with matched primary tumours (figure 2A–C). Western blot analysis of KPC tumours (n=5) and matched liver metastases (n=5) further corroborated these findings showing low expression of important stromal components such as fibronectin, SPARC and α-SMA in metastases (figure 2D). Healthy control liver tissue did not show immunoreactivity against such stromal components, whereas the epithelial marker E-cadherin was robustly expressed (figure 2D). As expected, normal liver tissue revealed very low amounts of activated fibroblasts and extracellular matrix components (figure 2A). MVD was analysed by CD31 immunohistochemistry and did not significantly differ between primary tumours (n=8) and liver metastases (n=8) (figure 2E).

**Figure 3:**
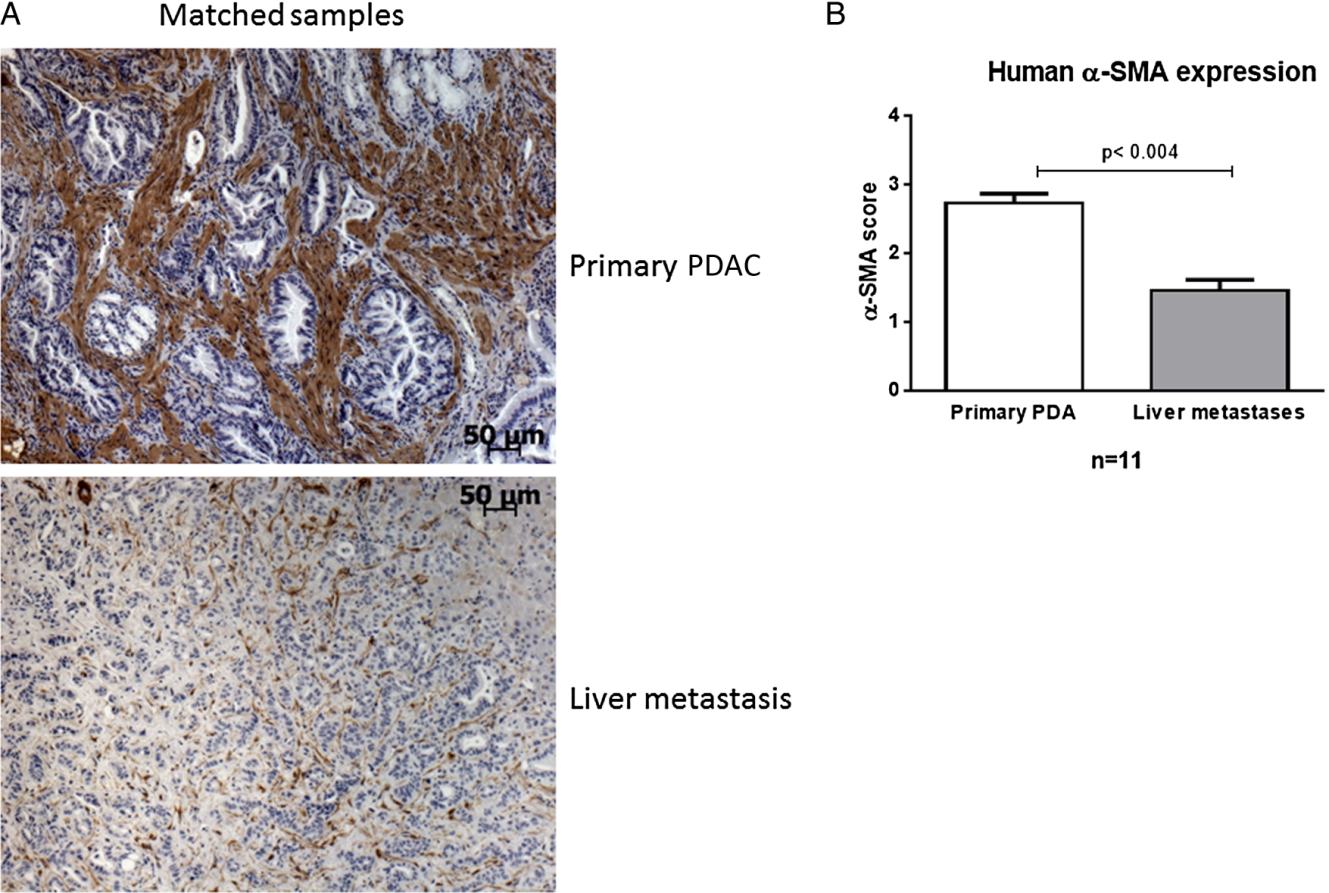
Human primary tumours reveal higher α-smooth muscle actin (α-SMA) content compared with matched liver metastases. (A) α-SMA immunohistochemistry of human primary pancreatic ductal adenocarcinoma (PDAC) with matched liver metastases of n=11 patients. Scale bars, 50 µm. (B) The α-SMA score was significantly higher in primary tumours compared with matched liver metastases (p<0.004; Wilcoxon matched-pairs signed-rank test).

To validate our findings in human specimen, we analysed a small cohort of patients with pancreatic cancer (n=11) with matched primary tumours and liver metastases. Although both tissues showed a clear desmoplastic reaction with heterogeneous distribution throughout the lesions, α-SMA-positive area was markedly reduced in all liver metastases compared with matched primary tumours (figure 3A, B).

Since the highest concentrations of gemcitabine were detected in primary pancreatic tumours, our results suggested that the stromal microenvironment may promote, rather than impede gemcitabine accumulation in bulk tissue.

### CAFs and PSCs show significantly elevated levels of intracellular gemcitabine in vitro

**Figure 4:**
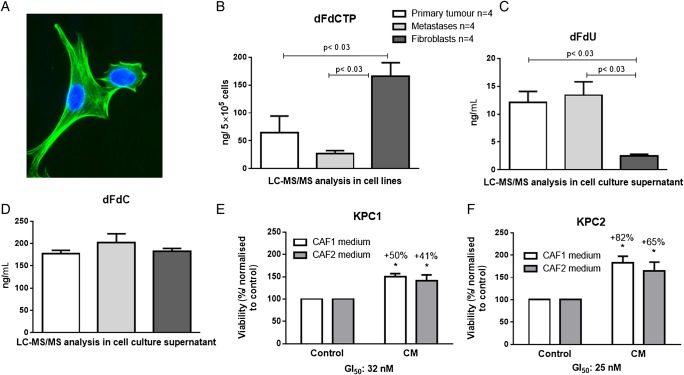
Fibroblasts accumulate activated gemcitabine while inactivation is decreased. (A) Typical morphology and α-smooth muscle actin immunoreactivity of cancer associated fibroblasts (CAFs) using immunocytochemistry. (B) Murine CAFs (n=2) and pancreatic stellate cells (PSCs) (n=2) as well as primary cell lines from KPC pancreatic tumours (n=4) and metastatic foci (n=4) were cultured and treated with 1 µM gemcitabine for 2 hours. Cell pellets and cell supernatants were subjected to liquid chromatography-mass spectrometry/mass spectrometry (LC-MS/MS) for analysis. 2′,2′-Difluorodeoxycytidine-5′-triphosphate (dFdCTP) was significantly increased in cell pellets from fibroblasts (PSCs and CAFs), compared with tumour cells (p<0.03). (C) The inactivated 2′,2′-difluorodeoxyuridine (dFdU) was significantly decreased in fibroblasts compared with tumour cells (p<0.03) indicating a greatly reduced gemcitabine inactivation in those cells. (D) Equal amounts of native gemcitabine (2′,2′-difluorodeoxycytidine (dFdC)) was detectable 2 hours after administration in cell culture supernatant. (E and F) 72 hours MTT assay with conditioned media (CM) of CAF1 and CAF2 preincubated for 24 hours with 30 nM gemcitabine in KPC1 (GI_50_ 32 nM) and KPC2 (GI_50_ 25 nM) cells shows significant increase in cell viability compared with CAF1 and CAF2 control media with fresh 30 nM gemcitabine prior to 72 hours treatment (KPC1—CAF1: p<0.002; KPC1—CAF2: p<0.04 and KPC2—CAF1: p<0.01, KPC2—CAF2: p<0.03; two-tailed, unpaired t-test).

Due to the observation that MVD did not correlate with intratumoural gemcitabine uptake, we hypothesised that the cellular components of the tumour would determine drug accumulation. Therefore, we generated primary cell lines from pancreatic tumours (n=4) and metastatic foci (n=4) from four different KPC mice. To dissect the stromal and neoplastic compartment, we further generated primary CAF lines (n=2) from murine pancreatic tumours as well as PSCs (n=3) from healthy B6 mice showing typical spindle-shaped morphology, and strong expression of α-SMA, SPARC and fibronectin (figure 4A and online supplementary figure S1B). The verification of CAFs was determined by PCR for the excised lox-stop-lox site of Kras^G12D^ (see online supplementary figure S1C). Following standardised treatment with 1 µM gemcitabine for 2 hours, cell pellets and supernatants were subjected to LC-MS/MS analysis. We found no significant differences in dFdCTP concentrations between CAFs (n=2) and PSCs (n=2) (see online supplementary figure S2A), and also no significant differences between tumour cell lines derived from primary tumours or metastatic lesions (figure 4B). However, we detected threefold to fivefold higher concentration of dFdCTP in fibroblasts (PSCs and CAFs) compared with neoplastic cells (p<0.03) (figure 4B). Gemcitabine and dFdU were below the level of quantification in all cells after 2 hours of treatment. In cell culture media, LC-MS/MS analysis revealed an approximately fivefold lower concentration of inactive dFdU from fibroblasts compared with supernatant from neoplastic cells (2.6 ng/mL, 95% CI 1.9 to 3.2 vs 12 ng/mL, 95% CI 5.7 to 18.4 for primary tumour cells, and 13.4 ng/mL, 95% CI 5.8 to 21.0 for metastatic tumour cells; p<0.03) (figure 4C). Notably, gemcitabine concentrations remained above 570 nM (150 ng/mL) in the media of all cell lines, and therefore were unlikely to be the limiting factor (figure 4D). To investigate whether this observation was specific for gemcitabine, we performed LC-MS/MS analysis for 5-FU in the same cell lines. Interestingly, we found no difference in 5-FU concentrations in the cell culture supernatant between the different cell types, and detected lower intracellular amounts of 5-FU in fibroblasts compared with various tumour cell lines (see online supplementary figure S2B, C). These data suggest that the intracellular concentration of dFdCTP is significantly higher in fibroblasts (CAFs and PSCs), when compared with neoplastic cells from primary tumours or metastatic lesions, and this effect was not observed for 5-FU suggesting drug metabolism rather than drug uptake as potential mechanism of action. As dFdCTP cannot cross the cell membrane, it is entrapped intracellularly in fibroblasts and is thus not available for tumour cells. And indeed, conditioned media of two CAF cell lines that were pretreated with gemcitabine for 24 hours at therapeutically relevant concentrations (GI_50_ of tumour cells) significantly increased tumour cell viability by 40%–80% in two KPC cell lines compared with CAF-conditioned media with fresh gemcitabine, suggesting a gemcitabine scavenging effect of CAFs in vitro (figure 4E, F).

### Gemcitabine inactivating genes are expressed at low levels in stromal cells in vitro and in vivo

**Figure 5:**
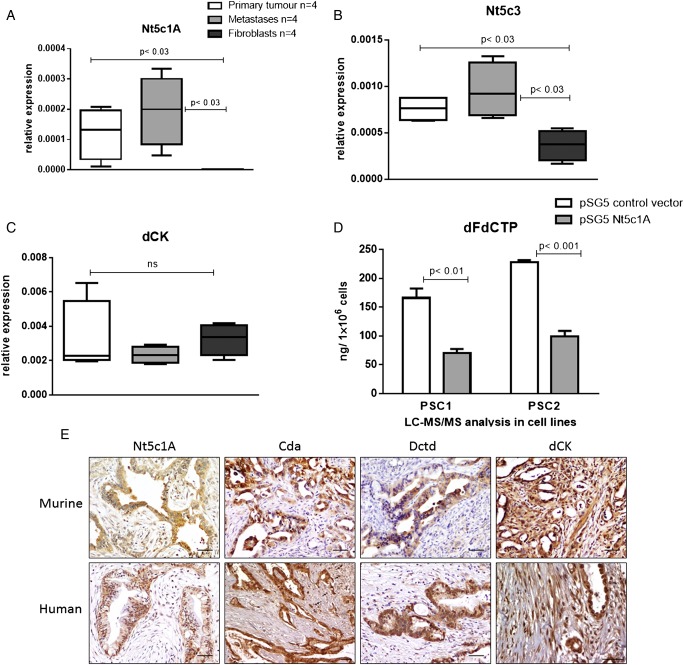
Gemcitabine inactivating genes are expressed at low levels in stromal cells in vitro and in vivo. RNA isolated from murine cancer associated fibroblasts (CAFs) (n=2) and pancreatic stellate cells (PSCs) (n=2) as well as primary cell lines from KPC pancreatic tumours (n=4) and metastatic foci (n=4) were subjected to quantitative reverse transcription-PCR. Gemcitabine metabolising enzymes were significantly downregulated in fibroblasts compared with tumour cells for (A) 5′-nucleotidase, cytosolic IA (Nt5c1A, p<0.03 and <0.03, respectively), (B) 5′-nucleotidase, cytosolic III (Nt5c3, p<0.03). (C) Deoxycytidine kinase (dCK) was not significantly different in fibroblasts compared with tumour cells (p=0.4); ns, not significant. (D) Gemcitabine treatment with 1 µM for 2 hours in PSC1 and PSC2 stably overexpressing Nt5c1A shows significant reduction of 2′,2′-difluorodeoxycytidine-5′-triphosphate (dFdCTP) (p<0.01 and <0.001, respectively, two-tailed, unpaired t-test). (E) Representative immunohistochemical pictures of murine and human tumour tissue showing cytidine deaminase (Cda), deoxycytidylate deaminase (Dctd) and Nt5c1A expression in tumour cells, whereas stromal cells (arrows) are almost completely devoid of immunoreactivity. dCK is robustly expressed in stromal cells (quantification see online supplementary figure S6). Scale bars, 50 µm.

Gemcitabine activation and inactivation is a complex multistep process that is driven by a number of enzymes and cellular transporters (see online supplementary figure S3). Reduced levels of dFdU in the supernatant of fibroblasts compared with tumour cells pointed towards the possibility that little gemcitabine inactivation may occur in these cells. Rapid metabolic inactivation of gemcitabine is predominantly catalysed by Cda or deoxycytidylate deaminase (Dctd).^30^ To investigate the mechanism of increased levels of dFdCTP in fibroblasts, we determined mRNA expression of a comprehensive panel of 15 genes involved in gemcitabine transport and metabolism (Ent1, Ent2, Cnt1, Cnt2, Nt5c1A, Nt5c3, Dctd, Rrm1, Rrm2, Rrm2b, Rpe, Rpia, TK2, dCK and Cda) in primary cell lines from pancreatic tumours, liver metastases and fibroblasts (PSCs and CAFs). Among these, only four genes were significantly downregulated in fibroblasts compared with cancer cells (Nt5c1A, Nt5c3, Rrm2 and Ent2) (figure 5A–C and online supplementary figure S4). Apart from low expression of Ent2 and Rrm2 that would not sufficiently explain high gemcitabine levels in fibroblasts,^31^ Nt5cA genes are a class of hydrolytic cytosolic 5′-nucleotidases that catalyse the hydrolysis of dFdCMP to gemcitabine. Low expression of Nt5c1A in fibroblasts may thus contribute to an increased pool of dFdCMP in fibroblasts that ultimately culminate in higher dFdCTP concentrations.^32^ To experimentally address this hypothesis, we stably transfected two murine PSC lines with Nt5c1A and treated with gemcitabine for 2 hours. Strikingly, LC-MS/MS analysis showed significantly reduced dFdCTP levels on Nt5c1A overexpression in both cell lines (figure 5D and online supplementary figure S5).

For in vivo studies, KPC mice were screened by high-resolution ultrasound prior to enrolment for appropriate tumour onset (6–9 mm diameter). Gemcitabine was administered at 100 mg/kg intraperitoneally every 3–4 days until end point. To assess gemcitabine metabolising proteins in murine and human PDAC, we performed immunohistochemistry of gemcitabine-treated KPC mice (n=24, median survival 12 days) and patient samples (n=50, median survival 15 months). Nt5c1A was strongly expressed in all murine and human tumour cells, whereas the tumour microenvironment was largely devoid of Nt5c1A expression (figure 5E). Cda and Dctd were not differentially expressed in fibroblasts and tumour cells at the mRNA level in vitro (see online supplementary figure S4), however, immunohistochemistry clearly demonstrated that Cda and Dctd were expressed at very low levels in stromal cells and robustly expressed in neoplastic cells in human and murine PDAC (figure 5E and online supplementary figure S6A, B). Notably, the main gemcitabine activating enzyme dCK was expressed in the stromal and neoplastic compartment of murine (n=24) and human PDAC (n=50) (figure 5E and online supplementary figure S6C, D). However, survival of mice and humans could not be correlated to stromal Cda, Nt5c1A and Dctd levels as the expression was very low in the stromal compartment in almost all cases. For dCK, high stromal expression did not correlate with significantly shortened survival in humans (14 months for high stromal dCK vs 16 months for low stromal dCK) and mice (15 days for high stromal dCK vs 11 days for low stromal dCK).

### Gemcitabine reduces metastatic burden in KPC mice

**Figure 6:**
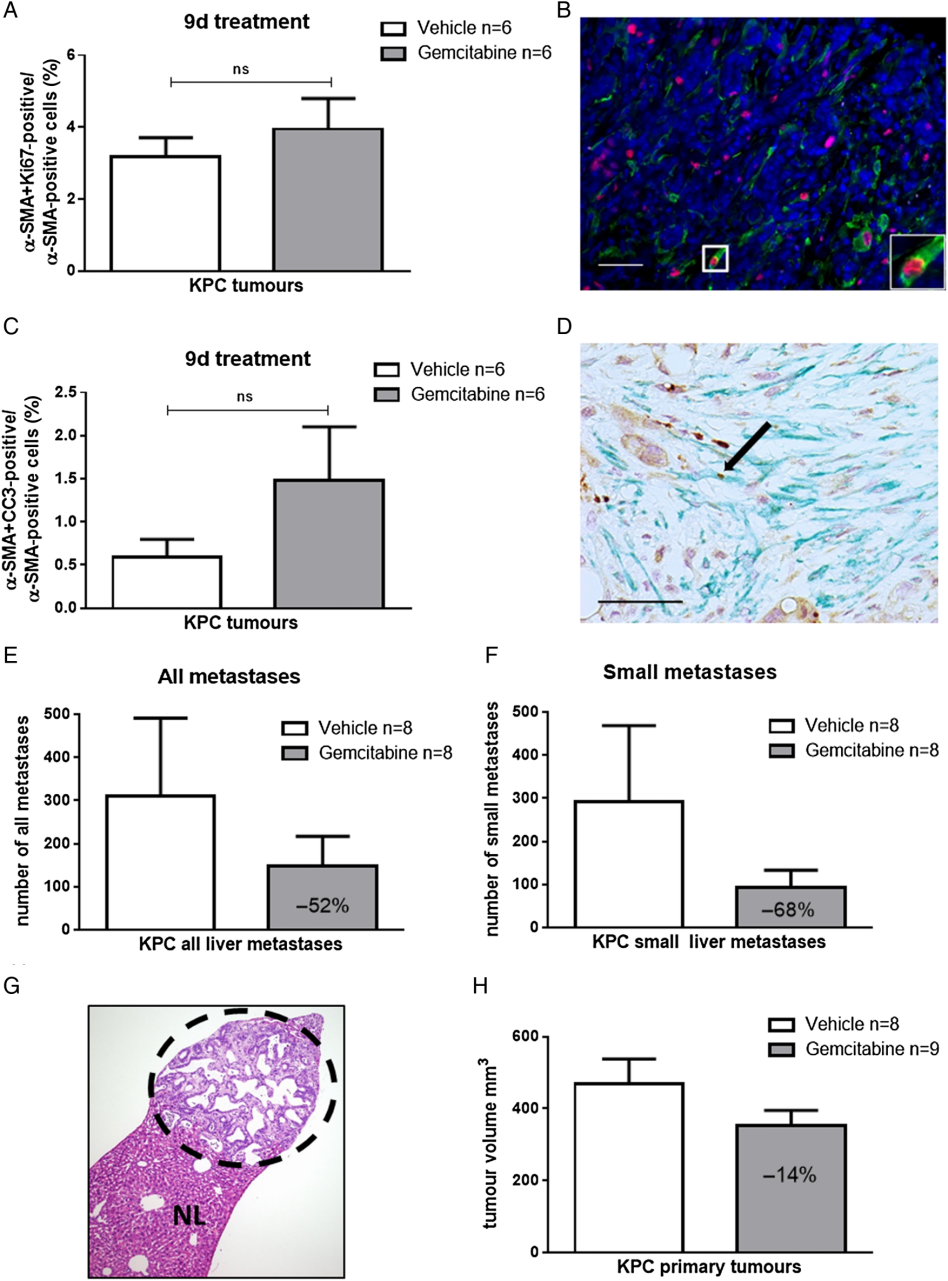
Gemcitabine treatment does not induce apoptosis in fibroblasts in vivo but reduces metastatic burden in KPC mice. (A and B) Archived tissue from primary pancreatic KPC tumours was evaluated retrospectively. Gemcitabine treatment had been administered every 3–4 days for 9 days. The last dose was given 2 hours prior sacrifice. Co-immunofluorescence (Ki67, red; α-smooth muscle actin (α-SMA), green) shows no significant difference in proliferation rate in α-SMA-positive cells after 9 days gemcitabine treatment (two-tailed, unpaired t-test), Scale bar, 50 µm. (C and D) Co-immunochemistry for α-SMA and CC3 does not show significant differences on gemcitabine treatment in KPC mice (two-tailed, unpaired t-test; arrow indicates apoptotic fibroblast). Scale bar, 50 µm. (E and F) Total number of all liver metastases and small liver metastases in 10 serial liver sections in a historical cohort of KPC mice treated with gemcitabine (n=8) or vehicle (n=8) for 9 days shows reduction of liver metastases by >50%. (G) Representative H&E of a small liver metastasis from KPC mouse (dotted circle) and normal liver tissue (NL). (H) Ultrasound volume measurements of corresponding mice reveals marginal response (−14%) on gemcitabine treatment for 9 days.

Taken together, these results suggest that low expression of several gemcitabine inactivating enzymes in fibroblasts results in increased concentrations of gemcitabine within the stromal compartment. To further characterise the cellular components of the tumour bulk, we analysed the number of α-SMA-positive fibroblasts and pan-cytokeratin-positive tumour cells in n=25 untreated KPC mice and found around three times more tumour cells (mean number 122/high power fields (HPF)) compared with fibroblasts (mean number 44/HPF, p<0.0001) (see online supplementary figure S7A, B). As human pancreatic tumours feature even more desmoplasia compared with GEMMs, and endothelial cells are often directly surrounded by fibroblasts and not tumour cells,^10^ it seems plausible that a significant number of gemcitabine metabolites may be trapped intracellularly in CAFs and thus are not available for tumour cells. Given the fact that stromal cells showed significantly higher concentrations of active gemcitabine compared with tumour cells (figure 4B), we asked whether the differential gemcitabine metabolism in tumour cells and fibroblasts results in increased gemcitabine sensitivity of fibroblasts in vitro and in vivo. CAFs are freely proliferative in vitro, and 72 hours 3-[4,5-dimethylthiazol-2-yl]-2,5-diphenyltetrazolium bromide; thiazolyl blue (MTT) assays showed comparable GI_50_ for gemcitabine (20–30 nM) in primary murine tumour cell lines (KPC n=2), tumour cells derived from metastatic foci (KPCm n=2) and fibroblasts (CAF n=2) (see online supplementary figure S8A, B). In vivo, we carefully analysed the proliferation rate of α-SMA-positive fibroblasts in archived tissue from a previously published preclinical trial.^28^ Co-immunofluorescence staining revealed a proliferation rate of 2%–5% in α-SMA-positive cells in vehicle-treated KPC tumours, and this rate did not change on gemcitabine treatment (figure 6A, B). Furthermore, co-immunohistochemistry for cleaved caspase-3 and α-SMA revealed a very low number of double positive cells indicating that CAFs are intrinsically resistant to gemcitabine in vivo (figure 6C, D). We speculate that the discrepancy between in vitro and in vivo chemosensitivity of CAFs is most likely due to the differences in proliferation rates, but that other factors such as prosurvival cues mediated between tumour cells and the microenvironment may also play a role.

Following our hypothesis that fibroblast scavenging is more prevalent in lesions with high fibroblast density, we aimed to test the response of gemcitabine on primary tumours and liver metastases in a historical cohort of KPC mice that had been treated with either vehicle (n=8) or gemcitabine (n=8) for 9 days.^28^ To this end, we quantified all liver metastases in 10 serial sections (each 40 µm apart) to assess the effects of gemcitabine on the overall frequency of metastases. Notably, we found a reduction of liver metastases by 50% following gemcitabine treatment, and this effect was most pronounced (68% reduction) when only small liver metastases were analysed (figure 6E–G). In contrast, corresponding primary KPC tumours were only reduced in volume by 14% on treatment confirming multiple previous reports from various groups that gemcitabine only mildly affects primary KPC tumours (figure 6H). When comparing the apoptotic rate of primary KPC tumours and corresponding liver metastases, we detected higher, albeit not significantly elevated levels of apoptotic tumour cells in liver metastases compared with primary tumours (see online supplementary figure S9A, B).

## Discussion

Despite the fact that gemcitabine is a highly active drug in vitro, monotherapy fails to show meaningful antitumour effects in patients with PDAC and genetically engineered mice. Apart from biochemical signalling loops within the tumour microenvironment,^33–38^ impaired drug delivery caused by stroma-rich and hypovascular tumours has been debated as one of the main reasons for the failure of anticancer agents in PDAC. However, antistromal approaches have failed in patients, and the increase in drug delivery may not necessarily imply that the anticancer compound is metabolically available and active against tumour cells. Indeed, a previous preclinical study conducted in our laboratory showed that systemic and intratumoural elevation of gemcitabine by cotreatment with a pharmacological inhibitor of gemcitabine inactivation (tetrahydrouridine) did not affect tumour volume or apoptotic rate in primary tumours.^28^

These considerations prompted us to reassess drug delivery in pancreatic cancer with a particular focus on the predominant cell types, that is, stromal cells and tumour cells. Our data show that gemcitabine accumulated in fibroblast-rich KPC tumours more effectively than in liver metastases displaying less desmoplasia and CAFs. This result was surprising as we had expected to see similar or higher levels of drug in liver metastases, which are surrounded by the well-perfused liver. In vitro, fibroblasts revealed a drug scavenging effect by metabolising gemcitabine thus entrapping significant amounts of dFdCTP that are not available for tumour cells anymore. Assuming that gemcitabine scavenging is more pronounced in bulk KPC tumours with strong desmoplasia, we investigated the effects of gemcitabine treatment on the frequency of liver metastases. On gemcitabine treatment, the number of all metastases was reduced by more than half, and this effect was particularly pronounced in small, only microscopically visible metastases. The effective killing of metastases and circulating tumour cells on gemcitabine has been recently proposed in a preclinical study. Metastatic lesions of all size were shown to have significantly higher apoptotic rates than primary tumours, and the metastatic burden was robustly reduced following long-term treatment with gemcitabine and nab-paclitaxel.^39^ The authors suggested that epithelial-to-mesenchymal transition in metastatic lesions may have promoted chemosensitivity towards nab-paclitaxel and gemcitabine. Thus, these observations together with our data may explain why gemcitabine affords a survival benefit in resected patients by targeting small metastases prior to clinical detection.

The reduced content of α-SMA fibroblasts in murine and human liver metastases compared with matched primary tumours is intriguing. Although the small set of matched human samples appear to confirm our murine data, confirmation from a larger set of human samples is required. Our observation is in contrast to previously published data that describe comparable stromal components in liver metastases and primary tumours.^40^ However, matched samples were only available in seven cases, and α-SMA staining was not performed for the matched dataset.^40^ Interestingly, a recent publication from the Stanger laboratory investigated the dynamic changes of the tumour stroma during progression from single metastatic cells to nanometastases, micrometastases, millimetastases and macrometastases in the KPCY mouse model. In line with our data, the authors reported reduced SPARC, collagen and α-SMA immunoreactivity in nanometastases, micrometastases and millimetastases.^39^ Although α-SMA staining was not performed for matched human metastases and primary tumours, the authors found that macrometastases became increasingly more desmoplastic and eventually recapitulated the primary tumour.^39^ In our study, we randomly used liver metastases for LC-MS/MS analysis but did not categorise lesions in several subgroups. Due to the small size of mouse liver metastases, we were also not able to simultaneously assess and correlate the stromal content and gemcitabine concentration in identical metastatic lesions.

Mechanistically, we found a number of gemcitabine metabolising enzymes with low expression in stromal cells that would explain the increased amount of gemcitabine and its activated metabolite. The two main gemcitabine inactivating enzymes are Cda and Dctd, and both act at different nodes during gemcitabine metabolism. Whereas Cda quickly inactivates gemcitabine to dFdU, Dctd metabolises dFdCMP to dFdUMP preventing further activation of dFdCMP to dFdCTP. To the best of our knowledge, differential expression of Nt5cAs in pancreatic cancer cells and fibroblasts has not been reported before. Since Nt5cAs catalyse the hydrolysis from dFdCMP to gemcitabine, it seems like the most suitable target for reprogramming the tumour stroma may be by decreasing dFdCTP concentrations in CAFs.

Furthermore, our results may also provide an explanation why the extent of desmoplasia was often reported to be negatively correlated with patient survival.^33^ ^40^ ^41^ As most of the previous prognostic datasets were derived from patients treated with gemcitabine, the high number of activated CAFs may have acted as a biomarker for poor drug response due to fibroblast drug scavenging.

Although fibroblast-depletion approaches may appear attractive for patients with PDAC to increase the intratumoural availability of gemcitabine for cancer cells, several studies recently reported that pharmacological and genetic depletion of activated fibroblasts results in accelerated pancreatic tumour growth, increased invasiveness, stemness and immune-modulation.^42–45^ Therefore, stromal and immunological reprogramming rather than CAF ablation is currently pursued as one potentially successful strategy to overcome therapy resistance.^45–49^ Our data further support this strategy showing that the metabolic programme of activated fibroblasts may lead to intratumoural drug redistribution and shift towards stromal cells rather than tumour cells. This drug scavenging effect of CAFs may be critical for therapeutic efficacy in pancreatic cancer, especially when several chemotherapies are combined. And indeed, we have recently shown that treatment with nab-paclitaxel increases intratumoural gemcitabine levels by reducing the levels of Cda protein in cancer cells through induction of reactive oxygen species-mediated degradation.^18^ This mechanism may partly explain the synergistic effect of this combination treatment in patients with pancreatic cancer.^5^

In conclusion, multiple studies suggest that impaired drug delivery is a major reason for the failure of therapeutic agents in pancreatic cancer. With our study, we add another layer of complexity showing that fibroblast drug scavenging increases intratumoural gemcitabine accumulation entrapping active gemcitabine within stromal cells thus making it unavailable for tumour cells. However, we cannot exclude the possibility that increasing drug delivery by modulating vessel patency and density might still have beneficial therapeutic effects in PDAC. Our data provide an alternative explanation for the failure of gemcitabine in PDAC and challenge the paradigm of a biophysical stroma barrier for gemcitabine delivery. Targeting the metabolic programme in CAFs may thus be a promising strategy to enhance the antiproliferative effects of gemcitabine in PDAC.
