# Supplementary material for: Fibroblast drug scavenging increases intratumoural gemcitabine accumulation in murine pancreas cancer
Source: Gut. 2017 Jan 10;67(3):497–507. doi: 10.1136/gutjnl-2016-311954 (PMC5868285; doi:10.1136/gutjnl-2016-311954)

Supplementary Material & Methods

Mouse strains

The LSL-KrasG12D, LSL-Trp53R172H, Pdx-1-Cre (KPC) mice have been described previously[1]. KPC mice develop advanced and metastatic pancreatic ductal adenocarcinoma with 100% penetrance at an early age recapitulating the full spectrum of histopathological and clinical features of human PDAC. Mice were housed at a 12 hr. light, 12 hr. dark cycle. All procedures were conducted in accordance to the institutional and national guidelines. Endpoint criteria for untreated controls (n=25) and gemcitabine treated mice (n=24) were defined as 20% body weight loss, general morbidity, lethargy, lack of social interaction or development of ascites. For co-immunofluorescence and dFdCTP/survival correlation, archived tissues from a previously published preclinical trial were used.[2] Here, gemcitabine had been administered at 100mg/kg by i.p. injection every 3-4d. Tissues were harvested 2h after the last gemcitabine administration. For expression analysis of Cda, dCK, Nt5c1A and Dctd, KPC mice (n=10) were treated with gemcitabine at 100mg/kg following high-contrast ultrasound screening using a Vevo2100 system. Enrolment criteria were defined at 6-9mm of tumour diameter as previously described[2]. The remaining n=14 KPC mice were obtained from 2 historical survival cohorts that were accordingly enrolled and treated with gemcitabine at 100mg/kg every 3-4 days.[2, 3]

Drug preparations

Gemcitabine powder (a 48% preparation of difluoro-deoxycytidine, dFdC) was provided by Addenbrooke's Hospital Pharmacy (Cambridge, UK) and resuspended in sterile normal saline at 10.2mg/ml. For in vitro experiments, gemcitabine hydrochloride (Sigma) and 5-FU (Sigma) were used.

Cell culture

Cell lines were derived from 4 murine KPC tumours and 4 metastatic foci (liver, spleen, ascites) as previously described,[1] and maintained in DMEM (Invitrogen) + 10% FBS (HyClone). CAFs were obtained by serial trypsinisation of small tumour pieces originating from *LSL-Kras$^{G12D/+}$;Ptf1a-Cre* (KC) mice and used at <10 passages. To distinguish CAFs from tumour cells, we performed PCR from genomic DNA for a single loxp site that is left after removal of the floxed stop cassette by Cre. The following primers were used: 5' ggg TAg gTg TTg ggA TAg CTg; 3' TCC gAA TTC AgT gAC TAC AgA TgT ACA gAg and the Jackslab protocol was used. Pancreatic stellate cells were purified from B6 mice (Charles River) by density gradient centrifugation as previously reported,[4] and immortalised by transfection with SV40 large T antigen (Addgene).

Co-Culture studies with conditioned medium

Primary murine CAFs were seeded with 1,000,000 cells per 10 cm dish and allowed to attach for 24 hours. Cells were treated for 24 hours with culture medium containing 30 nM gemcitabine or water as control. Cell viability was >80% at the time of medium collection compared to control. Subsequently, media were centrifuged at 1200 rpm for 3 min and supernatants were then used for 72h cell viability assay (MTT).

Cell viability assays - MTT

Tumour cell lines were seeded with 5,000 cells per well on a 96-well plate and were allowed to attach for 24 hours. Cells were then treated with conditioned medium. The control conditioned medium plus 30 nM fresh gemcitabine was used as control. For GI_{50} calculations the cells were treated with gemcitabine in concentrations between 0 nM and 40 nM. 72 hours after treatment, MTT reagent (Thiazolyl Blue Tetrazolium Bromide, Sigma) was added to the media with a

final concentration of 0.5 mg/ml and incubated for two hours at 37°C. Medium was carefully removed and precipitates were dissolved in 100 µl of acidified isopropanol. Absorption values were measured at 595 nm without reference wavelength (Autobio PHOmo Microplate reader, Labtec Instruments Co., LTD). Cell viability was expressed relative to controls.

LC-MS/MS of gemcitabine and 5-FU

For cell culture experiments, $5x10^5$ cells were cultured and treated with 1µM gemcitabine or 100µM 5-FU for 2h. Supernatant was collected and stored at -80°C. After trypsinisation, cell pellets were washed twice in ice cold PBS and stored at -80°C. All experiments were performed in triplicate.

RNA preparation

Pancreatic tissue samples were immediately placed in an RNA later solution (Qiagen) and stored for at least 24 hours at 4°C and then snap-frozen until processing. Total RNA was isolated using the Qiagen TissueLyser and Qiagen RNeasy kit. cDNA was synthesised from 1 µg of RNA using the Applied Biosystems QPCR cDNA Synthesis Kit (Applied Biosystems) and analysed by quantitative real-time PCR on a 272000410 Real-Time PCR system (Applied Biosystems) using relative quantification (ΔΔCt) with the Taqman gene expression assays (Applied Biosystems).

Stable Overexpression of Nt5c1A

Constructs for stable overexpression of Nt5c1A in murine pancreatic stellate cells were generated by amplification of the 1.1 kb coding region of *Mus musculus* 5'-nucleotidase, cytosolic IA (NM_001085502.1) according to the protocol recently published by Kari et al.[5]

In brief, the sequence was cloned into a derivative of the pSG5-vector containing a HA-Tag upstream an MCS separated from a Hygromycin resistance gene via a P2A sequence (pSG5-

HA-MCS-P2A-Hygro vector, a kind gift of S.A. Johnsen, University Medical Center Goettingen). The gene of interest was cloned between the NotI and NheI restriction sites following amplification using Phusion High-Fidelity DNA Polymerase (Thermo Fisher Scientific) and digestion with the respective restriction enzymes. The following primers were used at 5 pmol/µl: mNt5c1a Cl-NotI For 5'-GCTGACGCGGCCGCAATGGAACCAGGGC AGCCCCGGGAGGC and mNt5c1a Cl-NheI Rev 5'GCTGACGCTAGCCTGTGCACCTAA TGACTGCTTCGCAGCGGCAGCCCG. Correct insert sequence was confirmed by sequencing (primer 5'- CCTACAGCTCCTGGGCAACG). Plasmids were amplified, purified (NucleoBond® Xtra plasmid purification kit, Macherey-Nagel) and DNA concentrations were measured (Nano Photometer P330, INTAS). Linearised plasmids were generated using AseI restriction enzyme (Thermo Fisher Scientific) with subsequent phenol-chloroform purification. Immortalised pancreatic stellate cells were transfected with 6µg of linearised plasmids using TurboFect transfection reagent (Thermo Fisher Scientific). The vector without insert served as control. Cell selection started 48 hours post-transfection with 250 µg/ml hygromycin (Hygromycin B Gold, InvivoGen). Successful generation of stably transfected cell lines was confirmed by Western blot.

Protein extraction and Western blot analysis

Protein isolation from cell lines and tissue was performed as described before.[6] Western blot analysis was performed as described previously.[6] The primary and secondary antibodies are listed in **Supplementary Table 1**.

Histological examination

Tissues were fixed in 10% neutral buffered formalin (Sigma) for 24h and transferred to 70% ethanol. Tissues were embedded in paraffin, and 3-5µm sections were processed for H&E staining, immunohistochemistry and co-immunofluorescence using standard protocols as previously described.[7] Primary antibodies were incubated at 4°C overnight and are listed in **Supplementary Table 1**.

Methods of quantification

Automated quantification

Automated quantification of picrosirius and α-SMA staining area was conducted by using ImageJ applying a low threshold to account for background staining of necrotic tumour areas. Microvessel density was calculated by counting 10 HPF of independent 40x magnification pictures using ImageJ.

Manual quantification:

For co-immunofluorescence stainings 10 HPF at 40X were counted for each section. Stromal Cda and dCK immunoreactivity in human PDAC TMAs and KPC tumours was semi-quantitatively scored with 0= no positive staining, 1= few positive cells, 2= some positive cells, and 3= many positive cells. For metastasis quantification, 10 serial H&E liver sections, each 40µm apart, were quantified for metastatic burden. Stroma-epithelial cell ratio in untreated KPC mice (n=25) was manually quantified by counting all positive pan-cytokeratin and α-SMA positive cells in 5 HPF of identical regions of adjacent slides.

Immunohistochemistry, Immunocytochemistry and Immunofluorescence

Primary and secondary antibodies are listed in **Supplementary Table 1**. For immunocytochemistry cells were fixed with methanol and permeabilised with triton X-100. IC and IF slides were mounted with DAPI-containing mounting solution (Vectashield®).

Images were acquired on a Leica DMi8 microscope (Leica Microsystems CMS GmbH, Wetzlar, Germany). For IHC, remaining steps were carried out using appropriate Vectastain Elite ABC kits (Vector Labs) and DAB Peroxidase Substrate (Vector Labs), with haematoxylin counterstaining. The following reagents were used for special extracellular matrix stains: Picrosirius Red Stain Kit (Polysciences Inc.), and was performed according to standard protocol. Images were acquired using OLYMPUS DP27 camera and OLYMPUS cellSens Entry 1.12 software.

qRT-PCR FAM assays

For qRT-PCR FAM labelled assays were used in conjunction with TaqmanFast mastermix 96-wellplates. Actin was used as the endogenous control.

Actb	Mm00607939_s1
Cnt1	Mm01315355_m1
Cnt2	Mm04212034_m1
Ent1	mM00452176_m1
Ent2	mM00432817_m1
Dctd	Mm00618904_m1
Nt5c1a	Mm01192248_m1
Nt5c3	Mm0046604_m1
Cda	mM01341706_m1
dCK	Mm00432794_m1
Rrm1	Mm00485870_m1
Rrm2	Mm00485881_g1
Rrm2b	Mm01165702_gH
Tk2	Mm00445175_m1

Supplementary Table 1: Antibodies

**Name**	**Company**	**Catalogue number**	**Dilution**	**Application**
Hsp90	Cell Signaling	E289	1:5000	WB
α-SMA, Clone 1A4	Dako	M0851	1:1000/ 1:250/ 1:75/ 1:200	WB/ IHC (ms) + Co-IF/ IHC (h)/ IC
E-cadherin	Becton Dickinson	610181	1:2500	WB
Fibronectin	Abcam	ab2413	1:1000	WB
Nt5c1A	Assay Biotech	C15296	1:1000/ 1:300	WB/ IHC (ms, h)
HA-Tag	Cell Signaling	3724S	1:1000	WB
SPARC	R&D Systems	AF942	1:2000/ 1:100	WB/ IHC (ms)
Cda	Abcam	ab82346	1:300	IHC (ms)
Dctd	Elabscience	EPP12174	1:300	IHC (ms + h)
CD31	BD Pharmingen	553370	1:100	IHC (ms)
Pan cytokeratin	Abcam	ab6401	1:700	IHC (ms)
Ki-67 (SP6)	Thermo Scientific	RM9106-S0	1:200	IHC (ms)/ Co-IF
dCK	Abcam	Ab96599	1:100	IHC (ms)
Cda	LifeSpan Biosciences Inc.	LS-B10533	1:50	IHC (h)
dCK	LifeSpan Biosciences Inc.	LS-B10837	1:100	IHC (h)
CC3	Cell Signaling	9664L	1:100	Co-IHC
secondary HRP-antibodies	Dako	P0217/ P0449/ P0161	1:2000	WB
secondary antibodies: gt anti-ms/ dk anti-rb	Invitrogen - Alexa Fluor	A21131 (Alexa 488)/ A10042 (Alexa 568)	1:1000/ 1:500	IF/ IC
Secondary antibodies: gt anti-rb/ rb anti-ms	Dako	E0432/ E0354	1:300	IHC

Abbreviations: ms = mouse, h = human, gt = goat, rb = rabbit. WB: western blot, IHC: immunohistochemistry, IF: immunofluorescence

References:

1. Hingorani S. R., Wang L., Multani A. S., Combs C., Deramaudt T. B., Hruban R. H., *et al.* Trp53R172H and KrasG12D cooperate to promote chromosomal instability and widely metastatic pancreatic ductal adenocarcinoma in mice. *Cancer Cell*. 2005;**7**:469-83.
2. Neesse A., Frese K. K., Bapiro T. E., Nakagawa T., Sternlicht M. D., Seeley T. W., *et al.* CTGF antagonism with mAb FG-3019 enhances chemotherapy response without increasing drug delivery in murine ductal pancreas cancer. *Proc Natl Acad Sci U S A*. 2013;**110**:12325-30.
3. Cook N., Frese K. K., Bapiro T. E., Jacobetz M. A., Gopinathan A., Miller J. L., *et al.* Gamma secretase inhibition promotes hypoxic necrosis in mouse pancreatic ductal adenocarcinoma. *J Exp Med*. 2012;**209**:437-44.
4. Vonlaufen A., Phillips P. A., Yang L., Xu Z., Fiala-Beer E., Zhang X., *et al.* Isolation of quiescent human pancreatic stellate cells: a promising in vitro tool for studies of human pancreatic stellate cell biology. *Pancreatology*. 2010;**10**:434-43.
5. Kari V., Mansour W. Y., Raul S. K., Baumgart S. J., Mund A., Grade M., *et al.* Loss of CHD1 causes DNA repair defects and enhances prostate cancer therapeutic responsiveness. *EMBO Rep*. 2016.
6. Baumgart S., Chen N. M., Siveke J. T., Konig A., Zhang J. S., Singh S. K., *et al.* Inflammation-induced NFATc1-STAT3 transcription complex promotes pancreatic cancer initiation by KrasG12D. *Cancer Discov*. 2014;**4**:688-701.
7. Neesse A., Frese K. K., Chan D. S., Bapiro T. E., Howat W. J., Richards F. M., *et al.* SPARC independent drug delivery and antitumour effects of nab-paclitaxel in genetically engineered mice. *Gut*. 2014;**63**:974-83.

Supplementary figure legends:

Suppl Figure 1: Characterisation of murine cancer associated fibroblasts (CAFs)

A) LC-MS/MS analysis of bulk tumour tissue from n=10 KPC mice reveals no correlation between active gemcitabine (dFdCTP) and survival. **B)** Western blot analysis of cell lysates from primary CAFs (n=2), PSCs (n=3), and KPC tumour cells (n=4) showing high SPARC and fibronectin expression in stromal cells compared to epithelial tumour cells. **C**) PCR for a single loxp site that is left after removal of the LSL-KRAS G12D floxed stop cassette by Cre. CAFs (in duplicate) show only a short band (WT allele, 285bp) whereas tumour cells have a short and slightly larger band (mutant allele with loxp site, 325bp).

Suppl Figure 2: LC-MS/MS sub-analysis in CAFs and PSCs for dFdCTP

A) The triple phosphorylated active gemcitabine metabolite dFdCTP is not significantly different in CAFs (n=2 cell lines) versus PSCs (n=2 cell lines) 2 hours after addition of 1µM gemcitabine *in vitro* (two-tailed, unpaired t-test). Three samples were analysed per cell line. **B)** LC-MS/MS analysis of cell culture supernatant upon treatment with 100µM (13000 ng/ml) 5-

FU for 2hours. **C)** LC-MS/MS analysis of intracellular 5-FU upon treatment with 100 µM 5-FU. 5-FU was below the limit of quantification (BLQ), which was 0.4 ng/10^6 cells, in 2 of the 4 Mets and 3 of the 4 fibroblasts; for calculation purposes these were given the value of 0.4 ng/10^6 cells. Intracellular 5-FU was reduced in fibroblast cell lines (n=4) compared to cell lines from primary murine tumours and cell lines derived from metastatic foci.

Suppl. Figure 3: Gemcitabine pathway

Gemcitabine pathway demonstrates various steps of gemcitabine activation. The red arrows indicate gemcitabine inactivating genes that are downregulated in CAFs and PSCs, and may therefore contribute to fibroblast drug scavenging upon gemcitabine treatment.

Suppl. Figure 4: Expression analysis for gemcitabine transporters and enzymes

RNA isolated from murine CAFs (n=2) and PSCs (n=2) as well as primary cell lines from KPC pancreatic tumours (n=4) and metastatic foci (n=4) were subjected to qRT-PCR. * $p<0.05$.

Suppl. Figure 5: Recombinant expression of Nt5c1A in PSC1 and PSC2

Western blot analysis of PSC1 and PSC2 cells stably transfected with pSG5 control vector or Nt5c1A reveals robust overexpression of Nt5c1A. KPC tumour cell lysate served as positive control and shows comparable levels of Nt5c1A protein.

Suppl. Figure 6: Stromal expression of Cda and dCK in murine and human pancreatic cancer

A-D) Immunohistochemistry of Cda and dCK <was quantified in human pancreatic cancer tissues (n=50 patients) and KPC mice (n=24). Score 0: no expression, score 1: low expression, score 2: moderate expression, score 3: strong expression. Comparison of human and murine tissues shows a comparable stromal expression pattern.

Suppl. Figure 7: Epithelial to stromal cell ratio in KPC mice

A) Automated quantification using ImageJ for adjacent sections of pancreatic tumours in n=25 untreated KPC mice. Five 40x HPF were quantified per tumour and average cell numbers were blotted for epithelial cells (pancytokeratin) and fibroblasts (α-SMA) ($p<0.0001$, two-tailed,

unpaired t-test).**B**) Representative pictures of KPC tumours stained with pancytokeratin and α-SMA. Scale bars, 20µm.

Suppl. Figure 8: Growth inhibition of tumour cells and fibroblasts upon gemcitabine treatment *in vitro*

A+B) Primary KPC tumour cells (KPC1 and 2), KPC cells derived from metastatic foci (KPCm1-2) and CAFs (CAF1-2) were treated with 0, 10, 20, and 40nM gemcitabine for 72h. Subsequent MTT assay indicates GI_{50} values between 20-30nM gemcitabine.

Suppl. Figure 9: Gemcitabine induces apoptosis in liver metastases

A) Matched liver metastases and primary tumours from KPC mice (n=6) were assessed by immunohistochemistry for cleaved caspase-3 (CC3) (ns, two-tailed, paired t-test). **B**) CC3 immunohistochemistry of a primary KPC tumour (above) and a liver metastasis (below). Scale bars, 25 µm.

Suppl. Figure 1

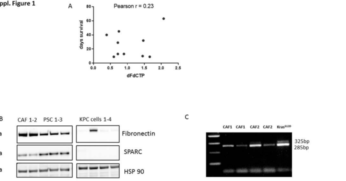

Suppl. Figure 2

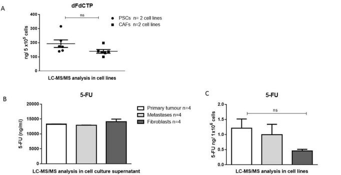

Suppl Figure 3

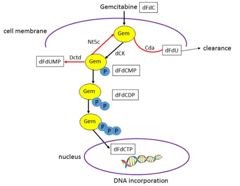

Suppl Figure 4

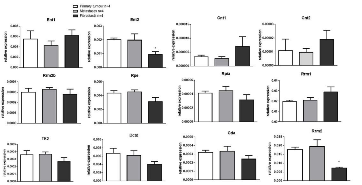

Suppl Figure 5

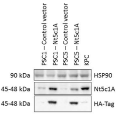

Suppl Figure 6

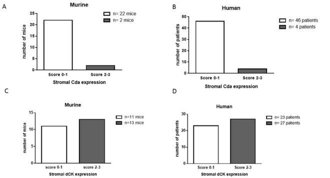

Suppl Figure 7

A

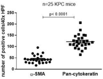

B

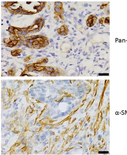

Suppl Figure 8

A

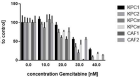

B

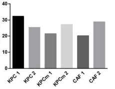

Suppl Figure 9

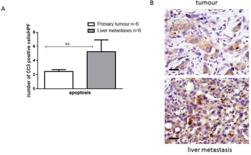

Supplement: supplementary material and methods [file gutjnl-2016-311954supp001.pdf]
